# In Silico Demonstration of Two-Dimensional Mass Spectrometry Using Spatially Dependent Fragmentation

**DOI:** 10.1021/jasms.2c00241

**Published:** 2023-02-06

**Authors:** Callan Littlejohn, Meng Li, Peter B. O’Connor

**Affiliations:** † ASCDT, Senate House, University of Warwick, Coventry, United KingdomCV4 7AL; ‡ Department of Chemistry, University of Warwick, Coventry, United KingdomCV4 7AL

## Abstract

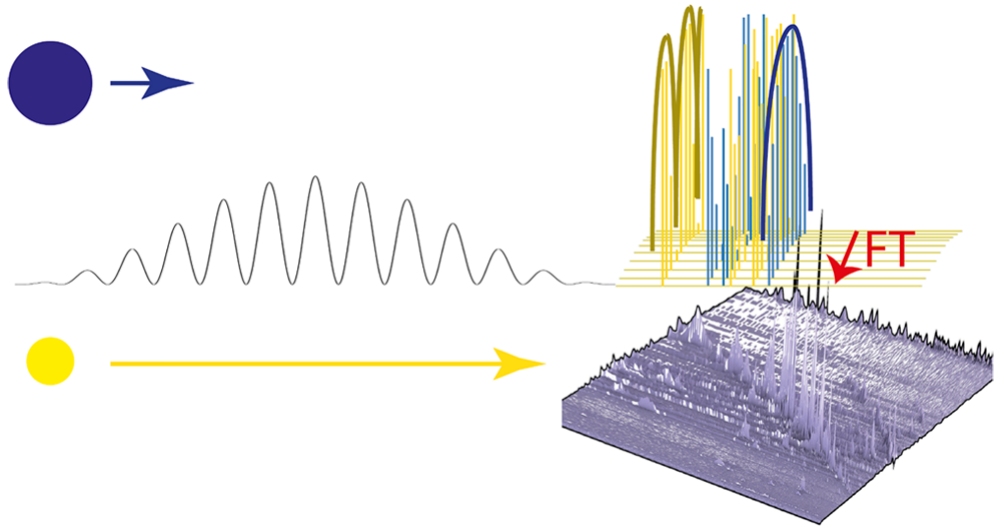

Two-dimensional mass spectrometry (2DMS) allows for the analysis of complex mixtures of all kinds at high speed and resolution without data loss from isolation or biased acquisition, effectively generating tandem mass spectrometry information for all ions at once. Currently, this technique is limited to instruments utilizing an ion trap such as the Fourier transform ion cyclotron resonance or linear ion traps. To overcome this limitation, new fragmentation waveforms were used in either a temporal or spatial configuration, allowing for the application of 2DMS on a much wider array of instruments. A simulated example of a time-of-flight-based instrument is shown with the new waveforms, which allowed for the correlation of fragment ions to their respective precursors through the processing of the modulation of fragmentation intensity with a Fourier transform. This application indicated that 2D modulation and Fourier precursor/fragment intensity correlation are possible in any case where separation, either temporally or spatially, can be achieved, allowing 2DMS to be applied to almost every type of mass spectrometry instrument.

Two-dimensional mass spectrometry (2DMS) is a data-independent tandem MS/MS analysis technique which allows the user to correlate fragment ions to their respective precursor ions through modulation in the fragmentation intensity. Evolving from the advances in 2D-NMR, 2DMS was first demonstrated in 1987 by Pfandler and Gäumann.^1^ At the time, data processing for 2DMS was an overwhelming task, but since then, it has been developed significantly along with advances in computing, allowing for more advanced denoising, processing, and peak picking algorithms.^2−5^ These advances have allowed for a large collection of applications to be developed, and 2DMS has been shown to be a useful tool in the analysis of agrochemicals, digested antibodies, and full proteins among other applications.^5−12^

2DMS is currently only applied in ion trap environments, with the most common implementation being the Penning trap using Fourier transform ion cyclotron resonance mass spectrometry (FTICR-MS). In the most common implementation, using the Gäumann pulse sequence, ions enter the cell and experience a low-voltage frequency sweep (a chirp) in order to excite them radially; after a delay, during which the ions continue to precess at their individual cyclotron frequencies, the ions are then subjected to a second low-amplitude chirp, called the “encoding” chirp as it causes the ions to separate radially depending on the phase of the ions relative to the pulse. This is dependent on the distance traveled which is defined by the cyclotron frequency of the ions along with their cyclotron radii. By incrementally increasing the delay between the first and second pulse between scans and taking many (256–8k) scans, precursor ions are effectively modulated through the central fragmentation zone of the trap at their own cyclotron frequencies, and the fragments are formed with their intensities also modulating at the precursor ion’s cyclotron frequency. The cyclotron frequency of the ions from theory is
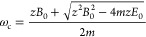
where *B*_0_ is the magnetic field, and *E*_0_ is a constant associated with the trapping voltage and the geometry of the cell.^13^

Stored waveform ion radius modulation (SWIM) is another method of performing a 2DMS experiment that allows the user to directly program the radial modulation, scan by scan, using the SWIM waveforms. Applied to a range of biologically and industrially relevant samples in the late 90s and early 2000s,^14−17^ this technique does not require careful control of the phase relationship of ions over several pulses and thus allowed for the development of a new type of 2DMS to be performed inside a linear ion trap.^18^ This application would allow for a wider array of instruments to perform 2DMS but is limited in that the instrumentation must be able to contain a linear ion trap (LIT). SWIM works using a stored waveform inverse Fourier transform (SWIFT) pulse sequence. In SWIFT, a waveform is produced in frequency space, and the inverse FT is applied to create a waveform for ions of specific frequencies to be excited to arbitrary levels defined by the requested frequency domain waveform. SWIM works by creating modulation waveforms using a waveform such as a sine wave as the initial starting waveform in frequency space and then between scans incrementing the nodes in the sine waveform so that different ions are excited to different, periodically varying radial extents for each waveform. When combined with a radially dependent fragmentation method, this approach modulates the precursors at their natural oscillation frequencies through the fragmentation zone, creating a 2D mass spectrum.

In a LIT, this dependency is correlated to the secular frequency of the ions in the ion trap, defined by
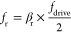
where *f*_r_ is the secular frequency, β_r_ is the stability parameter, and *f*_drive_ is the frequency of the RF voltage being applied to the quadrupole electrodes. Although in development, there is currently no applicatory work published using the SWIM method in a linear ion trap; however, its development and further use could increase accessibility of 2DMS.

Both methods raise a particular issue. Unless an ion trap is present, no 2DMS experiments can take place. This problem is not trivial, as the implementation of these techniques requires extensive tuning and preparatory work, and complex electronics and programs are required for this, which limits its use outside of an academic environment to date. If the technique is to reach a wider audience, it needs to encompass more types of mass analyzers.

While 2DMS is currently limited to ion-trap-based techniques, 2D correlation in this way is possible for all cases where separation of ions, temporally or spatially, can be achieved orthogonal (or partially orthogonal) to the axis of detection. Separation in this way is essentially the basis of all forms of mass spectrometry; therefore, all forms of MS should be able to achieve 2DMS with the right conditions.

The time-of-flight (TOF) mass analyzer is ubiquitous; the cheap and reliable nature of this technique allows for the technique to have a wide range of applications, including high resolutions at high mass ranges^19^ and space exploration.^20,21^ In TOF experiments, the ions are accelerated toward a detector using high-potential-difference electric fields. The speed of ions when traversing the field free time-of-flight region will be determined by the interactions of the charge on the molecule with the electric fields and the mass (inertia) of the ion with larger *m*/*z* appearing slower and smaller *m*/*z* appearing faster. Thus, when time from pulse to detection is measured, the mass to charge ratio can be determined using the equation:
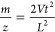
where *V* is the voltage on the repeller, *t* is the time taken, and *L* is the length of the flight tube. While an LIT can be added to the front end of a TOF instrument in order to perform 2DMS on resonantly excited ion packets using SWIM or the Gäumann pulse sequences, there is no current method of 2DMS which uses only spatial separation techniques, such as TOF.

TOF-MS is the best example of separation of ions in both a temporal and spatial case. If a temporal slice is taken, the spatial separation of ions will be very distinct in the lower *m*/*z* region while remaining much lower in the higher *m*/*z* region. Additionally, if a later time slice is taken, then the ions will have moved different distances dependent on their *m*/*z*; this relationship of reliable separations make 2DMS possible in a time-of-flight environment.

However, TOF is not the only spatial separation method possible. Electric sector and magnetic sector instruments also create a spatial separation of ion on a radial axis. This will also be shown to be useful in performing 2DMS.

This manuscript outlines some of the ways in which any spatial/temporal separation of ions can be used to perform two-dimensional mass spectrometry experiments.

## Simulation Methods

**Figure 1:**
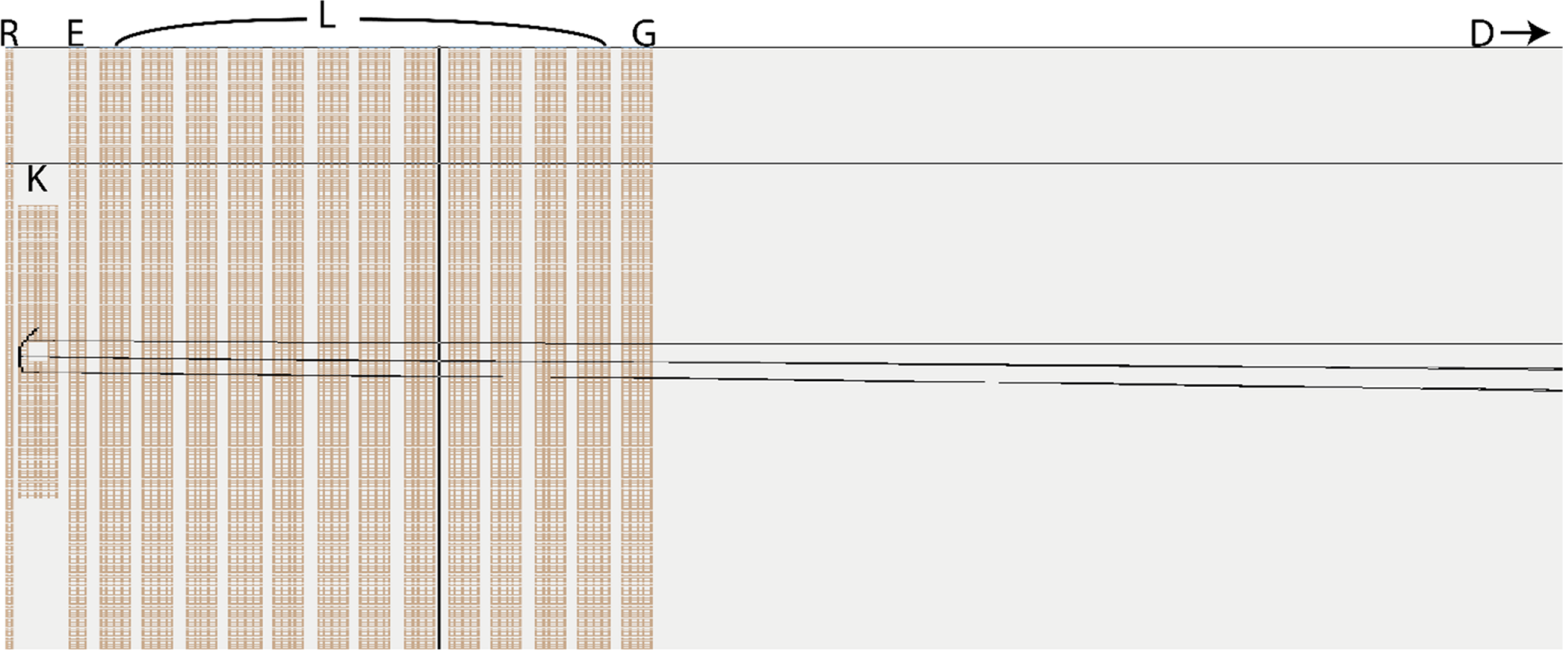
Overview of SIMION simulations for the TOF-based implementation. R represents the repeller, K, the side kick region, E, the extractor, L, a lensing region, G, ground, and D the detector.

All modeling was performed using SIMION 8.1, a particle trajectory simulation program distributed by Scientific Instrument Services.^22^ The simulations utilized an Intel Core i9 10900f, with typical usage of one core and 32 GB of RAM. In the case of the TOF variant, the workbench geometry was created to contain 15 electrodes with a source, extraction, and lensing region. The electrodes had apertures of 80 mm and were designed to be large enough that fringe fields would minimally perturb the ion channel. In SIMION, a split lens was produced in the TOF “pusher” region in the ion source to deflect the ions laterally, creating a spatial separation by *m*/*z*, which was followed by a patterned fragmentation zone so that 2DMS could be implemented. The workbench was operated at 0.5 mm/grid unit as shown in Figure 1. The ion optics stack was tuned in two parts; first, the post fragmentation optics state (the normal TOF operation) was tuned to reduce ion spread, as this could distort any results through differences in applied force and path difference. This was done using a user program feature within SIMION, which allows for operation of scripts, coded in Lua, which allow greater and more automated control of the workbench. The code changes the value of the voltage on the lens and extractor while keeping a steady gradient between each section and ground on the field-free region. After this lens stack was successfully tuned, the prefragmentation separation was then tuned, and it was found to be important that the kicked ion trajectory was as parallel to the detector as possible. It was also important that the side kick voltage was relatively low compared to the post side kick repelling voltage to allow for better separation across the split lens region.

**Figure 2:**
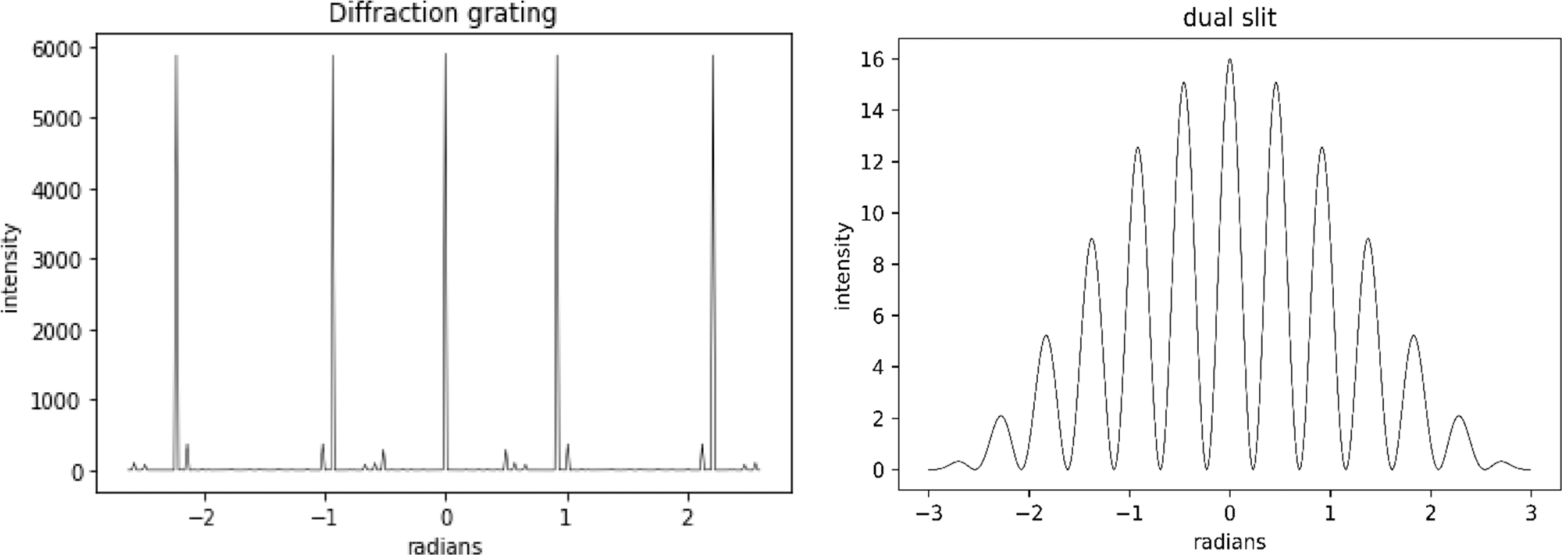
Examples of spatially dependent fragmentation patterns for 2DMS.

After the lateral kick, the precursor ions will be distributed spatially according to their time-of-flight. After a programmed delay, the ions are irradiated with a photofragmentation waveform, such as those shown in Figure 2, patterned in light/dark bands across the flight path of the ions so that ions with different TOF spatial positions will capture a photon with a probability that is proportional to the intensity of the light pattern at their position at the instant the light pulse pattern transits the dissociation cell, and the ions will subsequently fragment with a fragmentation efficiency dependent on their position. If the light pattern is then modulated over a series of experiments, or if the postside kick delay is incremented slowly, then the ions will fragment with a preprogrammed periodicity, which can be extracted by Fourier analysis. There are several ways to create the banded irradiation patterns needed, with some obvious examples being a dual slit, an interferometer, a simple diffraction grating, or programmed micromirrors.

The kick voltage was set at 7 V across the side kick, with a high voltage stabilizing field around the kick to keep the ion’s trajectory as parallel as possible to the detector. Post kick, the repeller was set to 1000 V, the extractor at 905, and the lens at 914. It is important to note that these values are low in comparison to many TOF techniques; however, for simplicity, this was ignored. In a real-world experiment, the higher voltages would be preferable, as they would further limit the impact of the sideways deflection geometry of the ion optics stack.

The experiment was set up to run 2^15^, or 32 768, scan lines with an incremental delay being chosen to allow for spatial separation of the ions in the kick region. The time step was determined at initialization to split evenly across the fragmentation zone.

The fragmentation zone was defined using the interference pattern expected when laser light is passed through a dual slit. This allowed for easier calculation of fragmentation intensity that was recorded each run at the ions’ position at the start of the fragmentation period and recorded in a CSV file using a workbench program. The fragmentation intensity was then plotted as a transient along the scan axis, and the Fourier transform was applied through a Python script using the NumPy implementation of the Cooley-Tukey FFT derived from the FFTPACK.^23−25^

**Figure 3:**
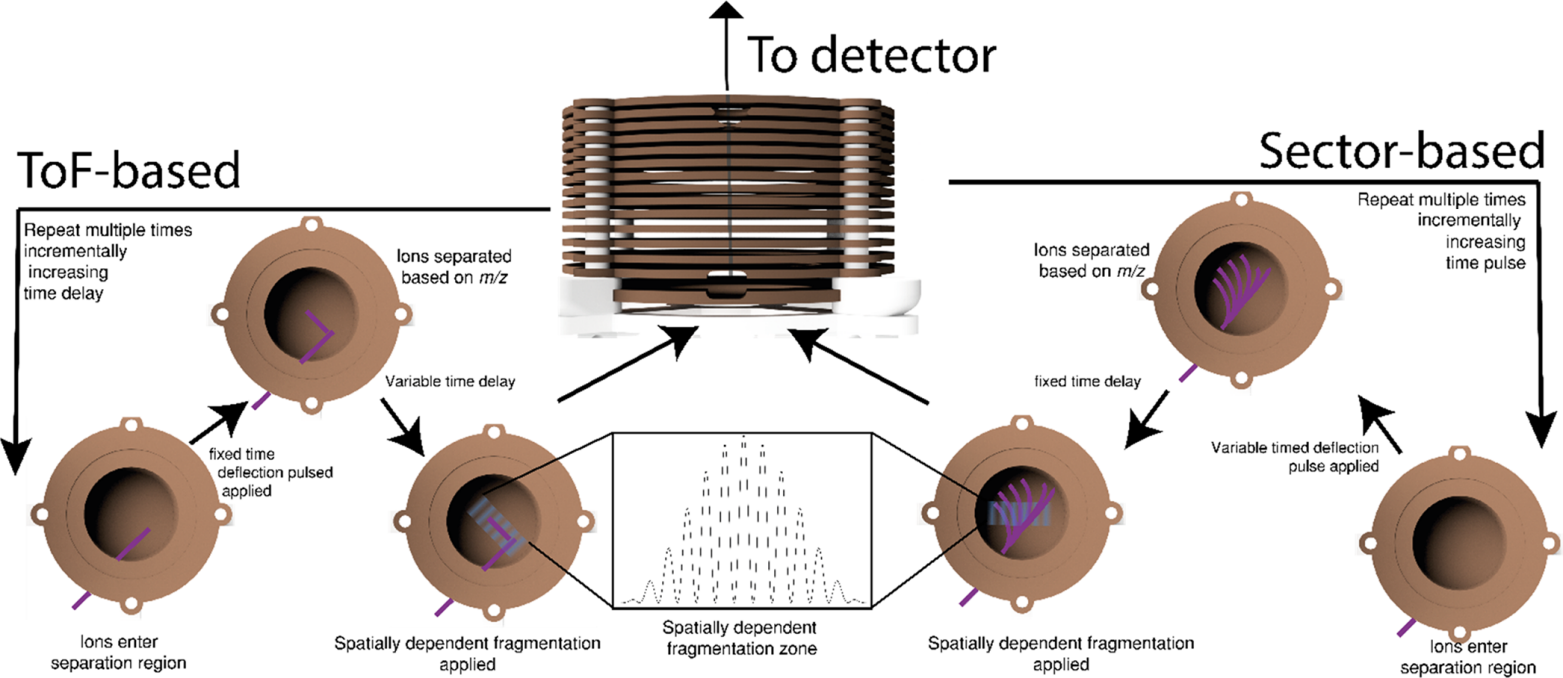
Step by step diagram of the two implementations shown within this work.

**Figure 4:**
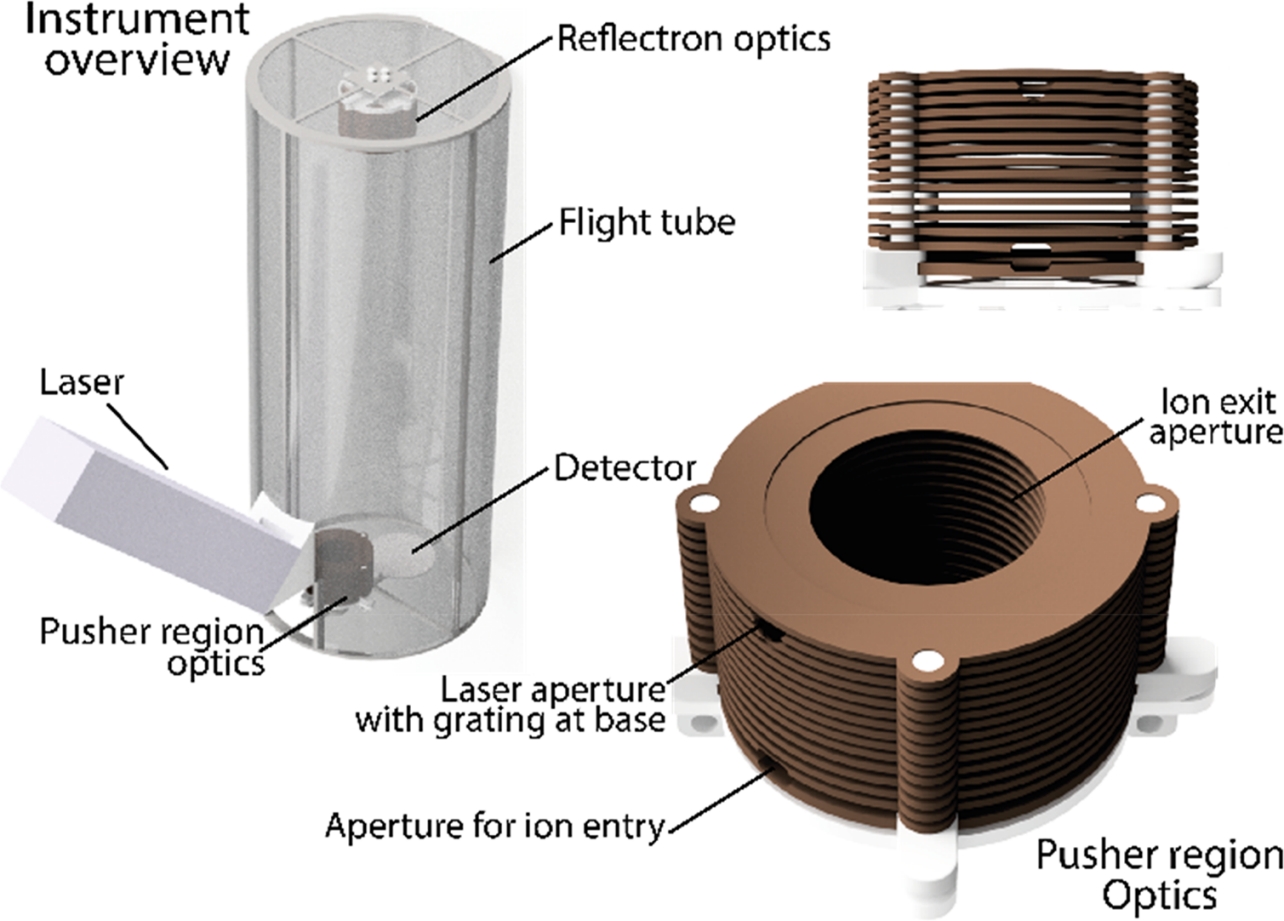
An example of a proposed instrument capable of performing 2DMS.

**Figure 5:**
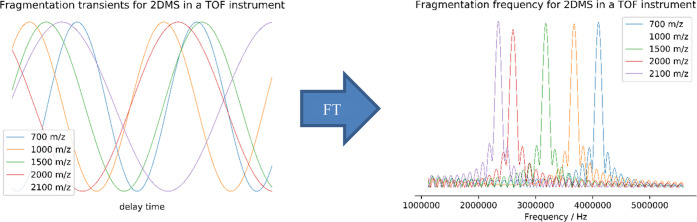
Sample of the transient detected through this 2DMS technique and its corresponding Fourier transform.

**Figure 6:**
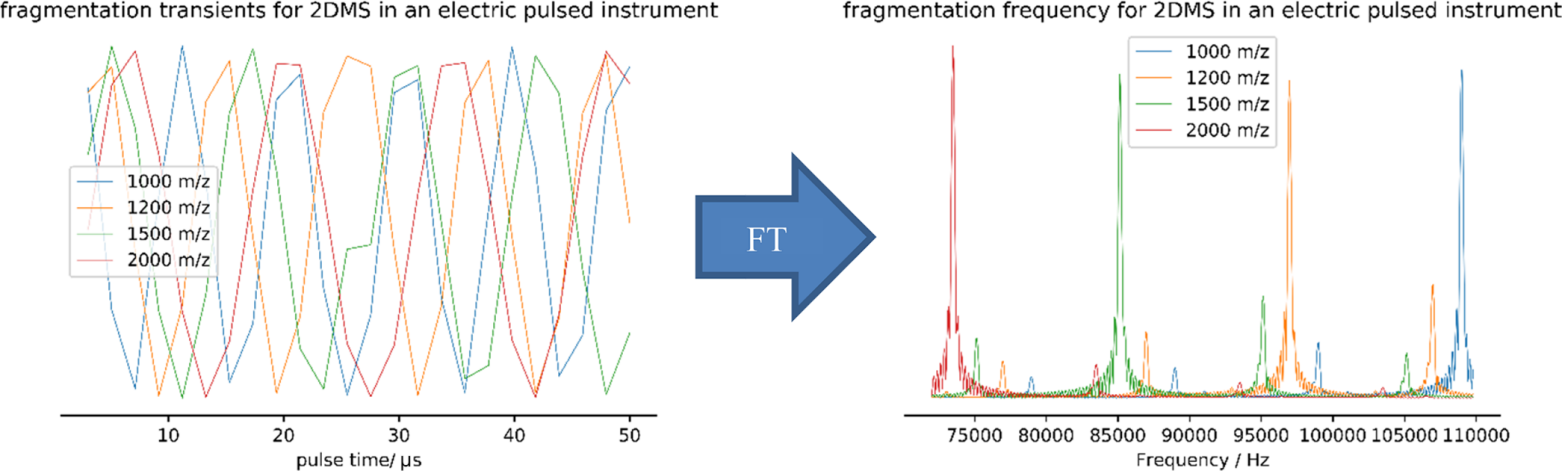
A sample of the transient detected through the electric pulse sector 2DMS experiment discussed being transformed into a frequency-based spectrum where frequency is dependent on precursor *m*/*z*.

Alongside the simulations of the TOF variant was the simulations of the electric sector variant. These simulations utilized the same PC but were performed at 0.1 mm/gu, and only the fragmentation zone was simulated, as this is the region of interest, and simulating the flight tube was demonstrated in the TOF variant. In this variant, a set voltage of 3 V was applied for a variable time on the ion’s entry into the fragmentation zone. This was simulated by defining in the geometry a block of conductive material at the ion entrance. The variable time was then stepped up by 1 μs each run, and this was allowed to progress for 2^13^, or 8192, runs. This was such that the time of activation was not longer than the pass-through time of the ions entering the fragmentation zone, which again, was simulated using a dual slit waveform but could easily be modified to incorporate other interface waveforms. Simplified schematics of this process can be found in Figure 3, and an example instrument is shown in Figure 4, and the corresponding FFTs of the observed peak intensity modulation are shown in Figures 5 and 6.

## Results and Discussion

The general description of this technique can be described as side kick-delay-fragment-detect. The ions first enter the split lens off-center and are kicked using the split lens. A variable delay then occurs with fragmentation using the spatially varying waveform. The ions are then detected using standard TOF instrumentation. By varying the delay, ions of different *m*/*z* will have traveled different distances across the fragmentation zone and thus will experience different fragmentation mode intensities. The fragmentation waveforms for each *m*/*z* will be different and directly correlated to the initial precursor *m*/*z* through a calibration equation that is derived as follows:
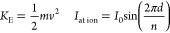
where *K*_E_ is the kinetic energy of the ion, which is linked to the initial voltage applied to the ions; as every ion with the same charge will be given the same *K*_E_, this ends up being constant where *m* is replaced with *m*/*z*. *v* is velocity, *I*_at ion_ is the photon fluence at specific locations, and *d* is the distance across the fragmentation zone. Finally, *n* is defined as the integer number of wave maxima present in the fragmentation zone.

*m*/*z* can be correlated to the delay time and distance using the equations:
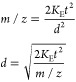
where *t* is the delay time. Furthermore, the fragmentation intensity can be estimated as a function of *m*/*z* and *t* by substituting *d* into the initial equation so that the equation becomes
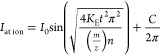
where *C* is a phase lag constant associated with the distance between the initial start point of the ions and the first maxima which can be discarded outside of absorption mode.

Thus, when combined with a laser irradiation pattern, as above, the fragment ion intensity at a specific *m*/*z* and time is determined by the precursor ion delay time. The frequency of the resulting wave becomes
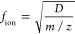
where:
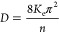


This calibration constant *D* is constant throughout the experiment for all masses. This means that ions will have an inverse square root relationship of frequency against *m*/*z*. This is interesting, as it means that many of the limitations seen in FT-ICR are also seen in this case. If the sampling rate is not at least twice the Nyquist rate, then unambiguous determination of the frequency will not be possible. In addition, the resolving power at high *m*/*z* (∼75 at 1000 *m*/*z* and ∼21 at 2300 *m*/*z*) is less than that of the lower *m*/*z*. This contrasts with normal TOF measurements where a lower *m*/*z* has worse resolution due to the rate of arrival at the detector. It is interesting that even though this device is technically a mini-TOF, because of the application of FT, some of the Fourier transform advantages will apply.

After performing a Fourier transform on the transients generated by the modulation in fragmentation intensity, it was found that indeed it was possible to link fragment mass to precursor mass through the fragmentation intensity frequency, which is the crucial prerequisite of 2DMS.

One obvious advantage of this approach is that it is limited primarily by the flight time of the ions and the repetition rate at which the laser irradiation pattern can be generated—both of which are expected to be in the 1 ms/1 kHz or better range. A major flaw with the current implementations of 2DMS is that the acquisition is slow, and therefore, it is difficult and time-consuming to adapt to external hyphenation such as ion mobility, LC, or GC. With the ability to scan fast, it may be possible to acquire a 2D mass spectrum for every mixture of compounds eluting within a chromatographic time slice of a few seconds’ width. The SIMION modeling herein shows that this approach is possible, but it will only be feasible after significant development including design of at least one new mass spectrometer, which will allow this lateral kick, delay, fragment, detect pulse sequence. Nevertheless, this instrument would open up applications of 2DMS to clinical and much more complex mixtures with minimal space charge and charge competition challenges compared to trapped-ion implementations of 2DMS.

The experiment is entirely tunable depending on the application. If a smaller mass range is necessary, then a much longer transient can be acquired, which can help improve precursor-ion resolving power. The delay time can be tuned to get better separation, which is trivial on modern electronics. Additionally, the geometry of the instrument can be designed to include some large ion optics to help increase the fragmentation area, further increasing the length of spatial axis used. Another way to increase the transient is to decrease the side kick voltage; however, this may cause instability in the ion trajectory, and additional instability may be incurred through the accuracy and speed of the DAC and the rise time of the voltages as they charge up the electrodes. The instrumentation is fully compatible with standard MS operation, as, by design, the ions enter the deflector at a region where, if deflected, will experience no adverse effects from the angular deflection.

This method may also hold benefits over FT-ICR. The benefit of being able to increase precursor ion resolving power by simply increasing TOF flight time range in the lateral kick dimension as well as the signal-to-noise increase that comes about due to the Felgett effect from use of the Fourier transform means that this technique in its TOF implementation could provide high-resolution peaks at higher intensity meaning more minute features and fragments in the spectrum could be accurately and precisely identified at a far greater speed than could ever be achieved in FT-ICR; additionally, as FT-ICR requires an FT of each individual scan as well as each data point in the scan dimension, the processing time of this implementation of 2DMS would be significantly improved over FT-ICR-2DMS.

This could be implemented in the first ToF region in a TOF-TOF, where the initial spread of ions is used as a much larger separation axis. It may also be possible to complete this in the first pass of a multireflecting TOF instrument.

While this is the most extensively modeled implementation, a few other notable examples should be considered. First, it is not entirely necessary to have the mini-ToF device inside the deflecting apparatus. If ions are injected at an angle as to make either tangential or chordial (off center) initial trajectory into the mass spectrometer, then using a split gate into the experiment, it may be possible to alter the ions’ positioning using electric pulses and to vary the pulse duration with a static fragmentation method. With a fixed pulse length, the kinetic energy to charge ratio will remain identical, and by imparting this force in a direction orthogonal to both the initial direction of travel as well as the final detection axis, it is possible to force the ions to travel at a different angular momenta. That is to say that the ions’ trajectory will be dependent on its *m*/*z*, as although the kinetic energy applied to each charge is identical, the mass difference means that the deflection will be greater with ions of smaller *m*/*z*.

2DMS using spatially dependent fragmentation zones in a time-of-flight may prove to be easier to implement into a commercial instrument, as there are fewer parts involved. In the case of the first shown implementation, the apparatus involved is a four-electrode sandwich, which can be difficult to build in practice, whereas this implementation would be simpler with a minimum of one extra electrode, which could take any reasonable form.

Where the first implementation is superior however is that it is far easier to glean the information from the spectrum. The spectrum appears to be significantly cleaner and devoid of noise because of the very linear excitation method; however, it is clear that the resolving power is a significant advantage in the electric sector variant with *y* axis resolving power of ∼900 RP_fwhm_ at 1000 *m*/*z* and ∼600 RP_fwhm_ at 2000 *m*/*z* after 4K scans, a roughly 10× increase in RP with 4× less scans. Due to the second method’s radial motion, extra variables must be accounted for. In this implementation, the calibration equation that must be built will be dependent on the time of activation. A suitable equation may be derived from the equation:
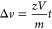
where Δ*v* is the change in speed in the direction of deflection, *V* is the electric field, and *t* is the pulse length. This assumes that the electric field will remain constant throughout the arc; however, this is a fair assumption at the correct input velocity, namely, where the ion ends up spiraling in or out of the electric field. This change in acceleration dictates the final exit angle from the electric sector. In such a way
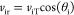
where *v*_ir_ is defined as the velocity in the orthogonal direction, *v*_T_ is defined as the total velocity, and θ_i_ is defined as the angle of entry to the radius at entry.

Thus, the final velocity (*v*_F_) can be determined by
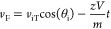


This velocity determines the final exit angle (θ_F_) of the ion through the electric sector through the equation:
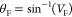


This is a simplification of the equation, as the forward force is not taken into consideration and indeed is set to 1 in this equation. This is fine, so long as the initial velocity is not too great as to overleap the electric field before the cut off event. The ejection angle leads to separation in a linear axis through the equation:
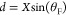
where *d* is the distance from the center of deflection and *X* is the total distance traveled after deflection. The total distance traveled after deflection will be reliant on *m*/*z*; however, given that the angle is also dependent, this should not affect the calibration in any great way. This leaves the final equation for final distance in the fragmentation axis at:
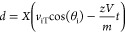


simplifying:
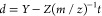
where:
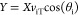
and:
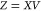


The final working for frequency would require the input of the all-important fragmentation waveform. Taking the same equation as before:
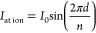


then, substituting for *d*:
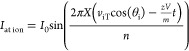
hence:
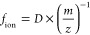
where:
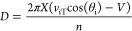


After substituting values for *v*_iT_ with the input velocity likely also coming from electric fields, it will also scale with *m/z.* The resulting relationship is
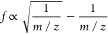
However, earlier it was stated that this method was not only applicable in time-of-flight instrumentation but every kind of mass spectrometer given sufficient modification. One such application was probed using a magnetic sector as a separation device followed by a detection method. While this inevitably leads to the same nature as one might find in the LIT where the method of 2DMS correlation is entirely separate to the method of 2DMS, correlation is entirely separate to the method of detection. It does allow for greater flexibility in the application of this technique. In this implementation, ions are sent toward the mass analyzer tangentially or chordially. They then pass through a small magnetic field, around 9 mT; this causes a spread to form with lower *m*/*z* ions being deflected more than higher *m*/*z* ions. The original technique of using a fragmentation waveform such as a diffraction grating, dual slit, or Fabry–Perot interferometer is then applied. Then, either the magnetic field can be altered by varying the current through an electromagnet or the fragmentation waveform can be changed through varying the position of the interference device. This is a far more difficult and slower way to implement this technique than the TOF implementations, though it is notable as an additional way of performing this type of 2DMS correlation without using an ion trap.

## Conclusions

In conclusion, through spatial fragmentation waveforms it is now possible to perform two-dimensional mass spectrometry experiments in a much wider array of instrumentation. The examples shown here are only a subset of instrument designs, in which this approach to 2DMS could be implemented; the simplicity and ubiquity in mass spectrometry for spatial or temporal separation mean that this technique is now possible throughout the full range of mass spectrometers.

Additionally, the spatial separation axis does not need to be entirely based in mass spectrometry. For example, it may be possible to use this device to link 2DMS to ion mobility spectrometry and imaging. In this technique, the *y* dimension is by definition dependent on the spatial separation axis. As ion mobility almost exclusively relies on spatial separation, this technique would be easily applicable.

This work has shown the feasibility of a simpler method of 2DMS without the need for an ion trap. Using spatially programmed fragmentation waveforms and timing systems, it is possible to perform 2DMS in a pure TOF-TOF type environment in addition to electric and magnetic sector preanalyzers. However, good precursor ion separation over a large mass range has been demonstrated here with full width half height resolving powers nearing 900 at 1000 *m*/*z*, which is large for a *y* dimension measurement. With the potential for much larger data sets to be pursued in the implementation of these techniques due to the increased speed, it may even be possible to reach systems where resolving power is reasonably high enough to perform the intricate analysis possible on the FTICR. However, it should be noted that the choice of final mass analyzer may influence the speed of aquisiton. While a standard TOF mass analyzer may be quicker, many more scans would be required to be averaged with the same incremental delay to obtain a statistical distribution of ions. An assumption has been made that the translational energy of the fragments relative to each other will be insignificant compared to the imparted kinetic energy from the pusher region. This limitation may be overcome through the use of a reflectron.^26^

Important in this work is the complete avoidance of any ion trap method, which up until now has been the only method of performing 2DMS (Penning trap and linear ion trap) This potentiates a larger catchment for potential 2DMS with the experiment now being possible on even the cheapest of mass spectrometers with reasonably little modification.

2DMS provides a powerful and often underutilized tool for analysis of complex mixtures, and this work will hopefully open up 2DMS to the masses.

## Supporting Information Available

Example SIMION GEM file; SIMION post kick tune script; ToF variant simulation workbench program; sector variant gem; sector-based simulation; comparison of FTICR, LIT, and spatial 2D (PDF)
