# Supplementary material for: In Silico Demonstration of Two-Dimensional Mass Spectrometry Using Spatially Dependent Fragmentation
Source: J Am Soc Mass Spectrom. 2023 Feb 6;34(3):409–16. doi: 10.1021/jasms.2c00241 (PMC9983000; doi:10.1021/jasms.2c00241)

SUPPORTING INFORMATION

In-Silico demonstration of two-dimensional mass spectrometry using spatially dependent fragmentation

Callan Littlejohn[1], Meng Li[2], Peter B. O'Connor[2*]

[1]ASCDT, Senate House, University of Warwick, Coventry, United Kingdom, CV4 7AL
[2]Department of Chemistry, University of Warwick, Coventry, United Kingdom, CV4 7AL
*corresponding author: p.oconnor@warwick.ac.uk

Example Simion GEM file

```
;Linear TOF 2D

;callan littlejohn

;25/10/2021


pa_define(2020,250,500,planar,y,elect,,0.5)


locate(0,0,125,1,-90);repeller

{electrode(100)

        {fill     {within  {cylinder(0,0,0,125,125,2)}

                   notin    {cylinder(0,0,-2,100,100,2)}}}}



;==================================================================

locate(5,0,125,1,-90);sidehit1

{electrode(2)

        {fill     {within  {cylinder(0,0,0,60,60,1)}

                   notin    {cylinder(0,0,0,30,30,1)}

                   notin    {box3d   (-3,0,0,125,125,-1)}}}}

locate(5,0,125,1,-90);sidehit2

{electrode(3)

        {fill     {within  {cylinder(0,0,0,60,60,1)}

                   notin    {cylinder(0,0,0,30,30,1)}
```

```
                    notin   {box3d  (3,0,0,-125,125,-1)}}}}
locate(3,0,125,1,-90);sidehitbackstabiliser
{electrode(1)
        {fill   {within {cylinder(0,0,0,60,60,1)}
                    notin   {cylinder(0,0,0,30,30,1)}}}}
locate(7,0,125,1,-90);sidehitstabiliser
{electrode(4)
        {fill   {within {cylinder(0,0,0,60,60,1)}
                    notin   {cylinder(0,0,0,30,30,1)}}}}

;==================================================================

locate(10,0,125,1,-90);extractor
{electrode(99)
        {fill   {within {cylinder(0,0,0,125,125,2)}
                    notin   {cylinder(0,0,0,30,30,2)}}}}
locate(14,0,125,1,-90);extlensi
{electrode(98)
        {fill   {within {cylinder(0,0,0,125,125,4)}
                    notin   {cylinder(0,0,0,90,90,4)}}}}
locate(20,0,125,1,-90);extlensii
{electrode(97)
        {fill   {within {cylinder(0,0,0,125,125,4)}
                    notin   {cylinder(0,0,0,90,90,4)}}}}

locate(26,0,125,1,-90);lensi
{electrode(96)
        {fill   {within {cylinder(0,0,0,125,125,4)}
                    notin   {cylinder(0,0,0,90,90,4)}}}}
locate(32,0,125,1,-90);lensii
{electrode(95)
        {fill   {within {cylinder(0,0,0,125,125,4)}
                    notin   {cylinder(0,0,0,90,90,4)}}}}
```

```
locate(38,0,125,1,-90);lensiii
{electrode(94)
	{fill	{within	{cylinder(0,0,0,125,125,4)}
			notin	{cylinder(0,0,0,90,90,4)}}}}
locate(44,0,125,1,-90);lensiv
{electrode(93)
	{fill	{within	{cylinder(0,0,0,125,125,4)}
			notin	{cylinder(0,0,0,90,90,4)}}}}
locate(50,0,125,1,-90);lensv
{electrode(92)
	{fill	{within	{cylinder(0,0,0,125,125,4)}
			notin	{cylinder(0,0,0,90,90,4)}}}}
locate(56,0,125,1,-90);lensvi
{electrode(91)
	{fill	{within	{cylinder(0,0,0,125,125,4)}
			notin	{cylinder(0,0,0,90,90,4)}}}}
locate(62,0,125,1,-90);lensvii
{electrode(90)
	{fill	{within	{cylinder(0,0,0,125,125,4)}
			notin	{cylinder(0,0,0,90,90,4)}}}}
locate(68,0,125,1,-90);lensviii
{electrode(89)
	{fill	{within	{cylinder(0,0,0,125,125,4)}
			notin	{cylinder(0,0,0,90,90,4)}}}}
locate(74,0,125,1,-90);lensix
{electrode(88)
	{fill	{within	{cylinder(0,0,0,125,125,4)}
			notin	{cylinder(0,0,0,90,90,4)}}}}
locate(80,0,125,1,-90);lensx
{electrode(87)
	{fill	{within	{cylinder(0,0,0,125,125,4)}
			notin	{cylinder(0,0,0,90,90,4)}}}}
locate(86,0,125,1,-90);lensxi
{electrode(86)
```

```
	{fill	{within	{cylinder(0,0,0,125,125,4)}
				notin	{cylinder(0,0,0,90,90,4)}}}}

locate(1000,0,125,1,-90);mcp
{electrode(0)
	{fill	{within	{cylinder(0,0,0,125,125,4)}}}}
```

Simion post kick tune script

```
--author callan Littlejohn
--date 25/10/2021
--version lens tune


simion.workbench_program()

--variables time

--main test voltages
adjustable min_lensvolts=850
adjustable max_lensvolts=850
adjustable min_extractorvolts=902
adjustable max_extractorvolts=910

--run numbers
adjustable run_numberlens=0
adjustable run_numberextr=0
adjustable max_trieslens=75
adjustable max_triesextr=10
adjustable request_rerun=1

--electrode variables
adjustable repevolts=0
```

```
adjustable extrvolts=0
adjustable lensvolts=0
adjustable _repel_voltage = 1000        -- repeller voltage
adjustable _extract_voltage = 905                      -- extractor (lens 1) voltage
adjustable _L2_voltage = 775                           -- lens 2 voltage
adjustable re_st1_voltage = 0       -- repeller-extractor stabil voltage
adjustable re_st2_voltage = 0       -- repeller-extractor stabil voltage
adjustable re_st3_voltage = 0       -- repeller-extractor stabil voltage

adjustable volt_step = 0     -- voltage step between test and 0V TOF
adjustable step_down_volt = 0
adjustable step_exlens_92 = 0               -- voltage step between extractor and lens
adjustable step_exlens_91 = 0               -- voltage step between extractor and lens
adjustable step_exlens_89 = 0               -- voltage step between extractor and lens
adjustable step_exlens_87 = 0               -- voltage step between extractor and lens
adjustable step_exlens_84 = 0               -- voltage step between extractor and lens
adjustable step_exlens_82 = 0               -- voltage step between extractor and lens

--spreads
adjustable spread01=0
adjustable spread02=0
adjustable spread03=0
adjustable spread04=0
adjustable spread05=0
adjustable spread06=0

--variables for spread calculations
adjustable yhit_max1=0
adjustable yhit_min1=0
adjustable zhit_max1=0
adjustable zhit_min1=0
adjustable yhit_max2=0
adjustable yhit_min2=0
adjustable zhit_max2=0
```

```
adjustable zhit_min2=0
adjustable yhit_max3=0
adjustable yhit_min3=0
adjustable zhit_max3=0
adjustable zhit_min3=0
adjustable yhit_max4=0
adjustable yhit_min4=0
adjustable zhit_max4=0
adjustable zhit_min4=0

--all the fun arrays yay
yhit_locations1={}
zhit_locations1={}
yhit_locations2={}
zhit_locations2={}
yhit_locations3={}
zhit_locations3={}
yhit_locations4={}
zhit_locations4={}
local update_pe=true
--might impliment later
adjustable spreads=4
adjustable max_ions=1000
adjustable mega_death_ray_parameter=9001

-- cleanup any old files.


local results_file = assert(io.open("120apet.csv", "w")) -- write mode
results_file:write("Welcome to the results file \n")
results_file:write("n, lens, extr, spread01  \n")


--lets get ready to rhumble
```

```
function segment.initialize ()
--i should probably put something in here but havent worked out what yet
sim_rerun_flym = request_rerun
end

function segment.fast_adjust()
        -- adjust fixed voltages 1st (repeller-extractor field)
        lensvolts=min_lensvolts+run_numberlens
        adj_elect100= _repel_voltage
        extract_voltage2 = _extract_voltage + run_numberextr
        adj_elect99= extract_voltage2

       volt_step= extract_voltage2-lensvolts/3
  step_down_volt= (lensvolts - (1*volt_step))
  adj_elect98= step_down_volt
       volt_step= extract_voltage2-lensvolts/3
  step_down_volt= (lensvolts - (2*volt_step))
  adj_elect97= step_down_volt

       adj_elect96= lensvolts

  -- adjust voltages between test and ground TOF plate
  volt_step= lensvolts/9
  step_down_volt= (lensvolts - (1*volt_step))
  adj_elect95= step_down_volt
  step_down_volt= (lensvolts - (2*volt_step))
  adj_elect94= step_down_volt
  step_down_volt= (lensvolts - (3*volt_step))
  adj_elect93= step_down_volt
  step_down_volt= (lensvolts - (4*volt_step))
  adj_elect92= step_down_volt
  step_down_volt= (lensvolts - (5*volt_step))
  adj_elect91= step_down_volt
  step_down_volt= (lensvolts - (6*volt_step))
```

```
    adj_elect90= step_down_volt
    step_down_volt= (lensvolts - (7*volt_step))
    adj_elect89= step_down_volt
    step_down_volt= (lensvolts - (8*volt_step))
    adj_elect88= step_down_volt
    step_down_volt= (lensvolts - (9*volt_step))
    adj_elect87= step_down_volt
end



function segment.other_actions()
    if update_pe then              -- if update flagged
        update_pe = false
        sim_update_pe_surface = 1       -- update the PE surface display
    end
end

function segment.terminate ()

if ion_number<=100 then
table.insert(yhit_locations1, ion_py_gu)
table.insert(zhit_locations1, ion_pz_gu)
end
if ion_number~=100 then return end

local lowest=1000
function findlowest(table)
lowest = 1000
for index , value in pairs(table) do
if value < lowest then
lowest=value
end
end
return lowest
```

```
end

local highest=-1000
function findhighest(table)
highest = -1000
for index , value in pairs(table) do
if value > highest then
highest=value
end
end
return highest
end
local spready1=0
local spready2=0
local spready3=0
local spready4=0
local spreadz1=0
local spreadz2=0
local spreadz3=0
local spreadz4=0
yhit_min1 = findlowest(yhit_locations1)
yhit_max1 = findhighest(yhit_locations1)
spready1 = yhit_max1-yhit_min1
zhit_min1 = findlowest(zhit_locations1)
zhit_max1 = findhighest(zhit_locations1)
spreadz1 = zhit_max1-zhit_min1
spread01 = ((spready1^2)+(spreadz1^2))^0.5


  results_file:write( run_numberlens ..
          ", " .. lensvolts                    ..
          ", " .. extract_voltage2 ..
```

```
                ", " .. spread01 ..                         "\n")
                                                results_file:flush()
run_numberlens = run_numberlens + 1

if run_numberlens==max_trieslens then

run_numberextr=(run_numberextr + 1)
run_numberlens=0
end
if run_numberextr==max_triesextr then
print("DONE!  See 1mmradius.csv.")

request_rerun=0
run_numberextr=0
end
yhit_locations1={}
zhit_locations1={}
yhit_locations2={}
zhit_locations2={}
yhit_locations3={}
zhit_locations3={}
yhit_locations4={}
zhit_locations4={}
end
```

ToF Variant simulation workbench program

```
--author: callan Littejohn
--Date: 30/10/2021
--version: full simulation

simion.workbench_program()
--laser constants
adjustable fragmentation_boundary=40
adjustable fragtime = 5
```

--scanning settings

adjustable scancount=75000

adjustable runperscan = 512

adjustable run_number=0

adjustable request_rerun=1

adjustable M1=300

adjustable M2=1000

adjustable M3=3000

adjustable p1=0

adjustable p2=0

adjustable p3=0

--kick settings

adjustable otherelect=50

adjustable repinkick=78--184--187

adjustable extinkick=87

adjustable kickstab1=126--215--220

adjustable kickstab2=101--225--220

adjustable kickside1=15--186

adjustable kickside2=8

adjustable kickto=115

adjustable set=0

adjustable tof300start=80

adjustable tof300end=100

adjustable tof300=0

--flight settings

adjustable repflight=1000

adjustable extflight=913

adjustable lenflight=894

```
--flight settings
adjustable repflight=1000
adjustable extflight=905
adjustable lenflight=914

--runvariables
adjustable step_down_volt=0
adjustable volt_step=0




local results_file = assert(io.open("massescollated.csv", "w")) -- write mode
results_file:write("Welcome to the results file \n")
results_file:write("t, 300 m/z, 500 m/z, 1000 m/z, 1200 m/z, 1500 m/z, 2000 m/z, 2300 m/z, 2500 m/z, 3000 m/z, \n")

local Mchange=0

function segment.initialize()
sim_rerun_flym = request_rerun
tof300=tof300start+((run_number/scancount)*(tof300end-tof300start))
end

function segment.fast_adjust()



set=1

-- flight optics in kick
if set==1 then
if ion_time_of_flight<tof300 then
	adj_elect100=repinkick
	adj_elect99=extinkick
	adj_elect98=otherelect
```

```
adj_elect97=otherelect
adj_elect96=otherelect
adj_elect95=otherelect
adj_elect95=otherelect
adj_elect94=otherelect
adj_elect93=otherelect
adj_elect92=otherelect
adj_elect91=otherelect
adj_elect90=otherelect
adj_elect89=otherelect
adj_elect88=otherelect
adj_elect87=otherelect
adj_elect86=otherelect
-- kick optics in kick
adj_elect1=kickstab1
adj_elect2=kickside1
adj_elect3=kickside2
adj_elect4=kickstab2
end
if ion_time_of_flight>=tof300 then

adj_elect100= repflight
adj_elect99= extflight

volt_step=(repflight-extflight)/4
step_down_volt= extflight+(3*volt_step)
adj_elect01= step_down_volt
step_down_volt= extflight+(2*volt_step)
adj_elect02= step_down_volt
step_down_volt= extflight+(2*volt_step)
adj_elect03= step_down_volt
step_down_volt= extflight+(1*volt_step)
adj_elect04= step_down_volt
```

```
	volt_step= extflight-lenflight/3
step_down_volt= (extflight - (1*volt_step))
adj_elect98= step_down_volt
step_down_volt= (extflight - (2*volt_step))
adj_elect97= step_down_volt

	adj_elect96= lenflight

-- adjust voltages between test and ground TOF plate
volt_step= lenflight/10
step_down_volt= (lenflight - (1*volt_step))
adj_elect95= step_down_volt
step_down_volt= (lenflight - (2*volt_step))
adj_elect94= step_down_volt
step_down_volt= (lenflight - (3*volt_step))
adj_elect93= step_down_volt
step_down_volt= (lenflight - (4*volt_step))
adj_elect92= step_down_volt
step_down_volt= (lenflight - (5*volt_step))
adj_elect91= step_down_volt
step_down_volt= (lenflight - (6*volt_step))
adj_elect90= step_down_volt
step_down_volt= (lenflight - (7*volt_step))
adj_elect89= step_down_volt
step_down_volt= (lenflight - (8*volt_step))
adj_elect88= step_down_volt
step_down_volt= (lenflight - (9*volt_step))
adj_elect87= step_down_volt
	step_down_volt= (lenflight - (10*volt_step))
adj_elect86= step_down_volt
	end
	end
	if set==0 then
	adj_elect100=repinkick
```

```
adj_elect99=extinkick
adj_elect98=otherelect
adj_elect97=otherelect
adj_elect96=otherelect
adj_elect95=otherelect
adj_elect95=otherelect
adj_elect94=otherelect
adj_elect93=otherelect
adj_elect92=otherelect
adj_elect91=otherelect
adj_elect90=otherelect
adj_elect89=otherelect
adj_elect88=otherelect
adj_elect87=otherelect
adj_elect86=otherelect
-- kick optics in kick
adj_elect1=kickstab1
adj_elect2=kickside1
adj_elect3=kickside2
adj_elect4=kickstab2
end

end

function segment.other_actions()
if ion_time_of_flight>=tof300 and ion_time_of_flight<=(tof300+fragtime)then
local p=50*sin(((ion_pz_gu*ion_pz_gu*2*math.pi)/10))+50
if ion_number==1 then
p1=p
end
if ion_number==2 then
p2=p
end
if ion_number==3 then
```

```
p3=p
end
if ion_number==4 then
p4=p
end
if ion_number==5 then
p5=p
end
if ion_number==6 then
p6=p
end
if ion_number==7 then
p7=p
end
if ion_number==8 then
p8=p
end
if ion_number==9 then
p9=p
end

-- if ion_number<=10 then
-- p1=p
-- if ion_number<p then
-- ion_mass=M1-100
-- end
-- end
-- if ion_number>10 and ion_number<=20 then
-- p2=p
-- if ion_number<(p+100) then
-- ion_mass=M2-200
-- end
-- end
-- if ion_number>20 and ion_number<=30 then
```

```
-- p3=p
-- if ion_number<(p+200) then
-- ion_mass=M3-500
-- end
-- end
-- if ion_number>30 and ion_number<=40 then
-- p4=p
-- end
-- if ion_number>40 and ion_number<=50 then
-- p5=p
-- end
-- if ion_number>50 and ion_number<=60 then
-- p6=p
-- end
-- if ion_number>60 and ion_number<=70 then
-- p7=p
-- end
-- if ion_number>70 and ion_number<=80 then
-- p8=p
-- end
-- if ion_number>80 and ion_number<=90 then
-- p9=p
-- end
-- Mchange=1


end end


function segment.terminate()
if ion_number == 9 then
  results_file:write( tof300 ..
        ", " .. p1                    ..
      ", " .. p2                      ..
```

```
", " .. p3 ..
                    ", " .. p4 ..
                    ", " .. p5 ..
                    ", " .. p6 ..
                    ", " .. p7 ..
                    ", " .. p8 ..
                    ", " .. p9 .. "\n")
        run_number=run_number+1
        if run_number>scancount then
        request_rerun=0
        end end

end
```

Sector variant gem

```
;sector based 2d
;callan Littlejohn
;15/02/2022

pa_define(120,1500,1500,planar,none,elect,,0.1)

locate(0,75,75,1,-90);repeller
{electrode(100)
        {fill   {within {cylinder(0,0,0,75,75,2)}
                        }}}
locate(5,70,10,1);nudge1
{electrode(1)
        {fill   {within {centered_box3d(0,0,0,2,3,3)}}}}

locate(5,80,10,1);nudge1
{electrode(2)
        {fill   {within {centered_box3d(0,0,0,2,3,3)}}}}

locate(8,75,75,1,-90);repeller
```

```
{electrode(99)
    {fill    {within  {cylinder(0,0,0,75,75,2)}
                       notin    {cylinder(0,0,0,50,50,2)}}}}
```

Sector based Simulation

```
-- callan littlejohn
-- lua code for the sector based 2D
-- 15/02/2022
simion.workbench_program()

adjustable surround_voltage_start=20
adjustable kick1=19
adjustable kick2=20
adjustable start_kick_incriments=0.002
adjustable start_time=10
adjustable request_rerun=1
adjustable run_counter=0
adjustable max_runs=25000
adjustable tof300=26
adjustable fragtime=2
adjustable stated=0
local results_file = assert(io.open("massescollated.csv", "w")) -- write mode
results_file:write("Welcome to the results file \n")
results_file:write("t, 300 m/z, 500 m/z, 1000 m/z, 1200 m/z, 1500 m/z, 2000 m/z, 2300 m/z, 2500 m/z, 3000
m/z, \n")

function segment.initialize ()
sim_rerun_flym = request_rerun
end

function segment.fast_adjust ()
adj_elect100=surround_voltage_start
adj_elect99=surround_voltage_start
if ion_time_of_flight>start_time and ion_time_of_flight<start_time+(run_counter*start_kick_incriments)+1
then
```

```
adj_elect01=kick1
adj_elect02=kick2
else
adj_elect01=surround_voltage_start
adj_elect02=surround_voltage_start
end end
function segment.other_actions()
        if ion_time_of_flight>=tof300 and ion_time_of_flight<=(tof300+fragtime)then
        local p=50*sin(((ion_pz_gu*ion_py_gu*2*math.pi)/10))+50
        if ion_number==1 then
        p1=p
        end
        if ion_number==2 then
        p2=p
        end
        if ion_number==3 then
        p3=p
        end
        if ion_number==4 then
        p4=p
        end
        if ion_number==5 then
        p5=p
        end
        if ion_number==6 then
        p6=p
        end
        if ion_number==7 then
        p7=p
        end
        if ion_number==8 then
        p8=p
        end
        if ion_number==9 then
```

```
            p9=p
            end


            end
            if stated==0 then
            if ion_pz_gu>75 then
            print(ion_time_of_flight)
            stated=1
            end end end
function segment.terminate()


if ion_number == 9 then
     results_file:write( run_counter+1 ..
                ", " .. p1                                  ..
               ", " .. p2                                   ..
               ", " .. p3 ..
                                  ", " .. p4 ..
                                  ", " .. p5 ..
                                  ", " .. p6 ..
                                  ", " .. p7 ..
                                  ", " .. p8 ..
                                  ", " .. p9 .. "\n")
            run_counter=run_counter+1
            end
if run_counter>max_runs then
request_rerun=0
end
end
function segment.tstep_adjust()
ion_time_step = 0.1 -- X usec
end
```

Comparison of FTICR, LIT, and Spatial 2d

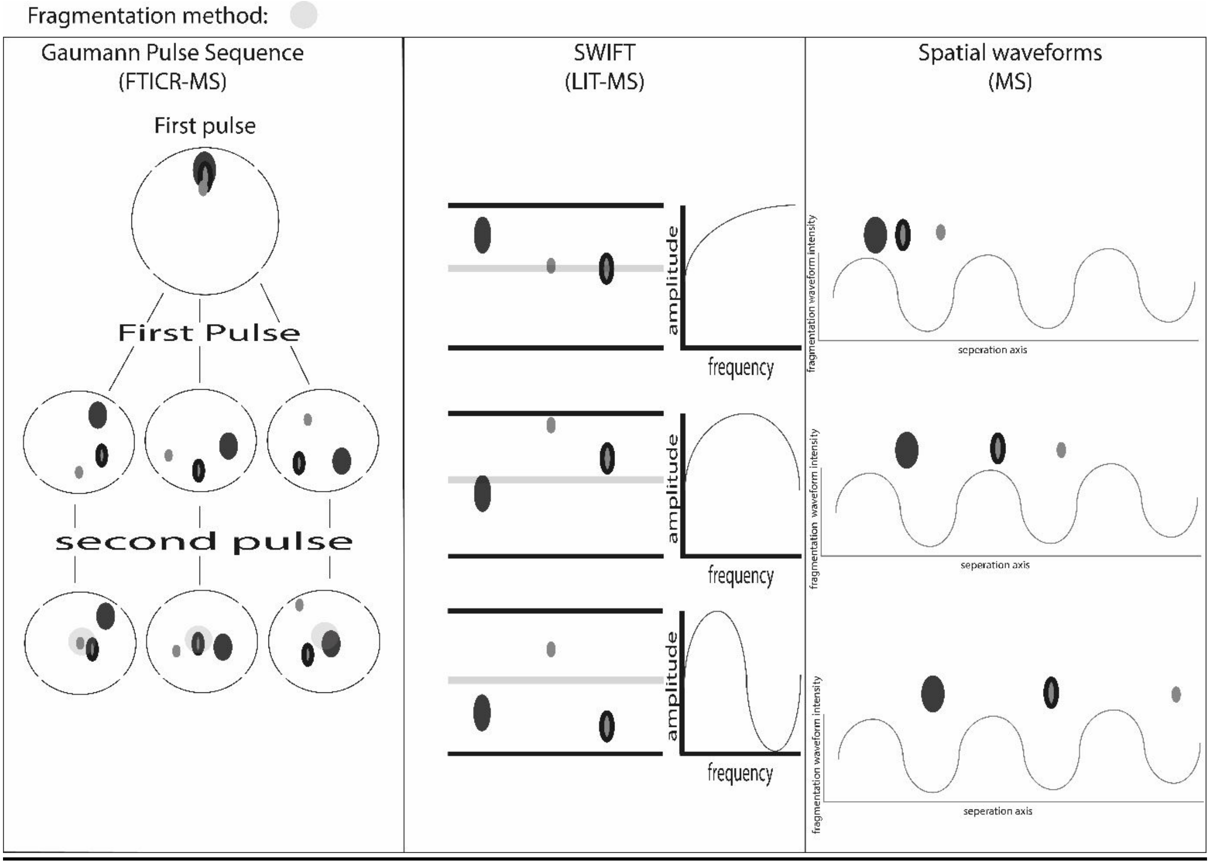

Seperation in tof

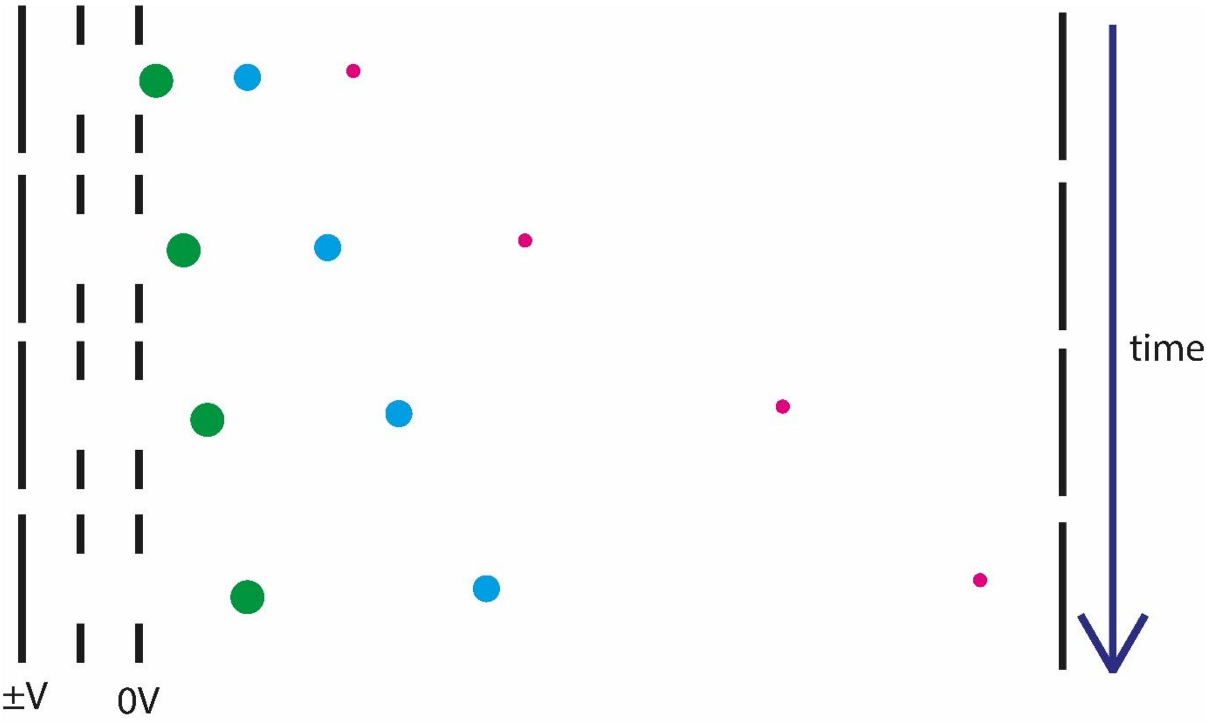

Sector like seperation

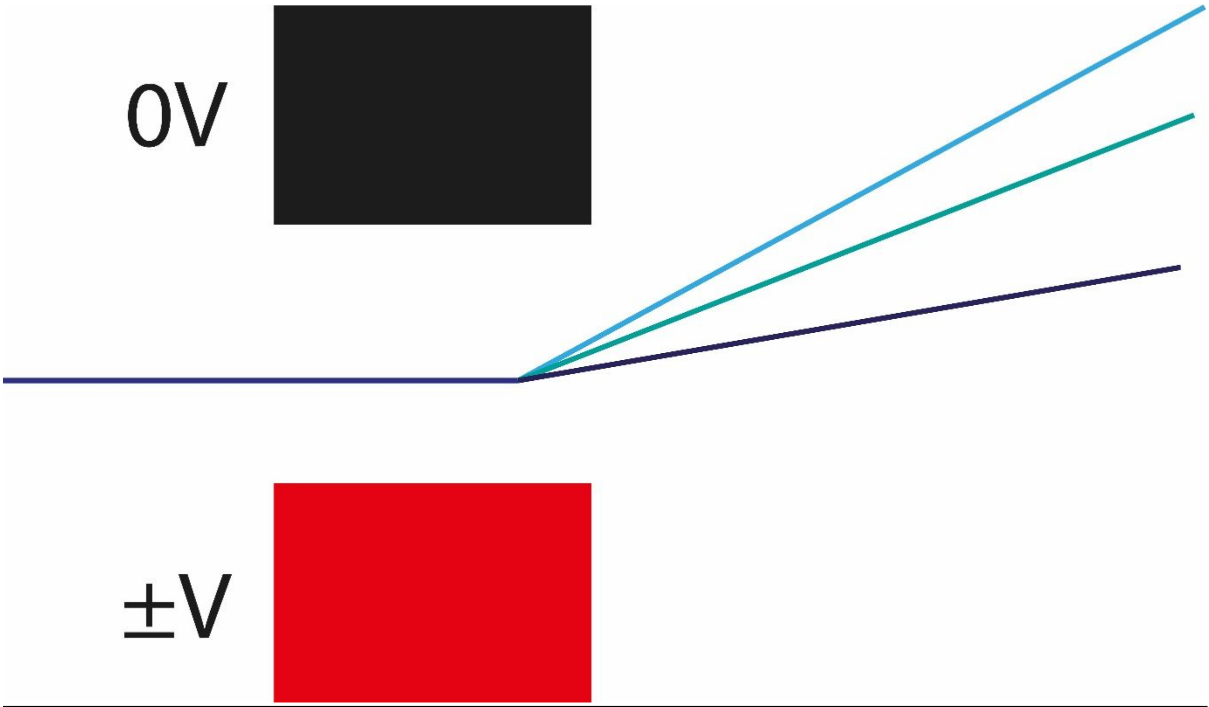

2D pulse program for FTICR

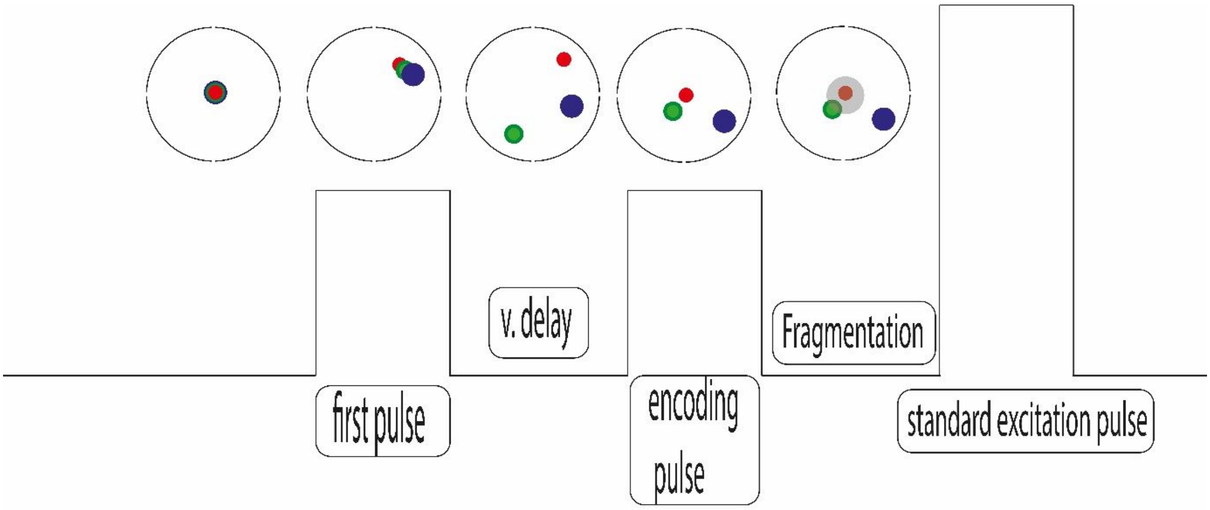

Supplement: Supplementary file 1 — js2c00241_si_001.pdf [file js2c00241_si_001.pdf]
